# Passivity-Based Adaptive Hybrid Synchronization of a New Hyperchaotic System with Uncertain Parameters

**DOI:** 10.1100/2012/920170

**Published:** 2012-12-27

**Authors:** Xiaobing Zhou, Zhangbiao Fan, Dongming Zhou, Xiaomei Cai

**Affiliations:** ^1^School of Information Science and Engineering, Yunnan University, Kunming, Yunnan 650091, China; ^2^Bureau of Asset Management, Yunnan University, Kunming, Yunnan 650091, China

## Abstract

We investigate the adaptive hybrid synchronization problem for a new hyperchaotic system with uncertain parameters. Based on the passivity theory and the adaptive control theory, corresponding controllers and parameter estimation update laws are proposed to achieve hybrid synchronization between two identical uncertain hyperchaotic systems with different initial values, respectively. Numerical simulation indicates that the presented methods work effectively.

## 1. Introduction

 Hyperchaos, characterized as a chaotic attractor with more than one positive Lyapunov exponent, was first reported by Rössler [1]. Due to its great potential in theoretical and engineering applications, hyperchaos has been investigated extensively over the past three decades. Since the hyperchaotic Rössler system was reported, many more hyperchaotic systems have been proposed, such as hyperchaotic Chua's system, hyperchaotic Chen system, and hyperchaotic LC oscillator system.

**Figure 1:**
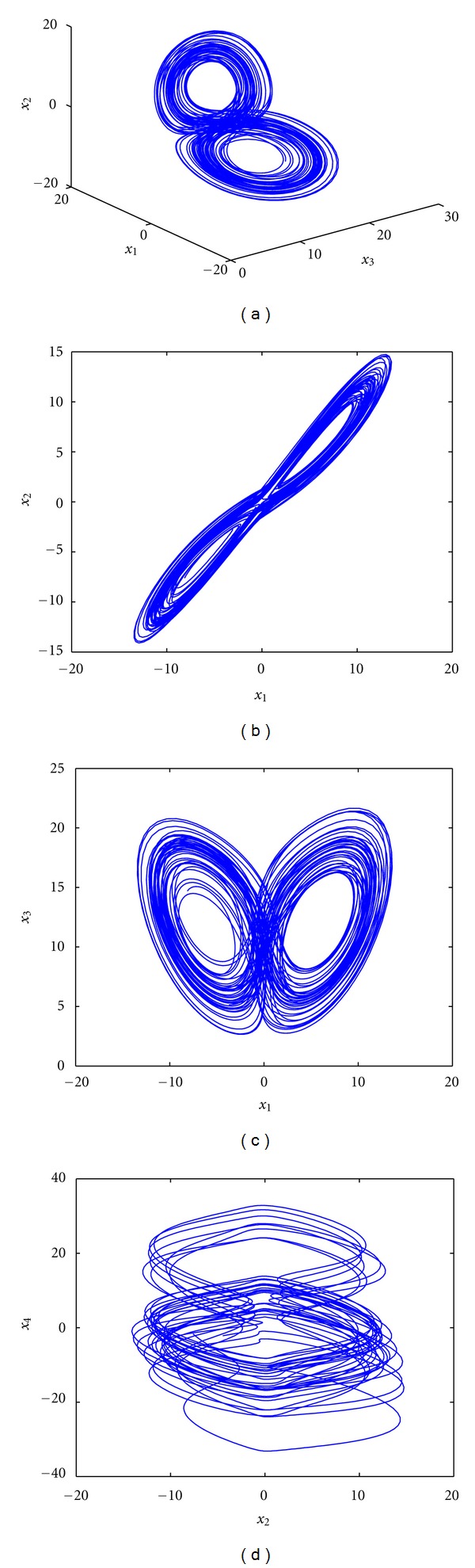
Hyperchaotic attractor for system (1).

Very recently, the authors [2] constructed a new 4D hyperchaotic system by adding one state variable into the 3D Lü chaotic system. The new hyperchaotic system is shown in the following form:

(1) $$\begin{array}{c} {\dot{x}}_{1}=a\left(x_{2}-x_{1}\right)+x_{4},\\ {\dot{x}}_{2}=cx_{2}-x_{1}x_{3},\\ {\dot{x}}_{3}=-bx_{3}+x_{1}x_{2},\\ {\dot{x}}_{4}=dx_{1}+kx_{2}x_{3}, \end{array}$$

where *x*_1_, *x*_2_, *x*_3_, and *x*_4_ are state variables; *a*, *b*, *c*, *d*, and *k* are system parameters, respectively. System (1) is dissipative and has only one equilibrium point (0,0, 0,0). When *a* = 35, *b* = 3, *c* = 12, *d* = 1, and *k* = 0.5, system (1) exhibits a hyperchaotic attractor, which is illustrated in Figure 1. 

In recent years, chaos/hyperchaos synchronization has attracted increasingly attentions due to its potential applications in the fields of secure communication and optical, chemical, physical, and biological systems, and so forth [3–5]. Until now, a wide variety of approaches have been proposed for the synchronization of chaotic/hyperchaotic systems, such as linear or nonlinear feedback control [6], delayed feedback control [7], adaptive control [8], backstepping design [9], and sliding mode control [10], just to name a few. Among all kinds of synchronization schemes, hybrid synchronization, which has been proposed by Li [11], is a noticeable one. In hybrid synchronization scheme, the complete synchronization and antisynchronization coexist in the system. So, to apply hybrid synchronization to communication systems, the security and secrecy of communication can be enhanced greatly [12].

Nowadays, the concept of passivity for nonlinear systems has aroused new interest in nonlinear system control. By applying the passivity theory, Yu [13] designed a linear feedback controller to control the Lorenz system. Wei and Luo [14] proposed an adaptive passivity-based controller to control chaotic oscillations in the power system. In [15, 16], Kemih realized chaos control for chaotic Lü system and for nuclear spin generator system, respectively. In [17], Wang and Liu also applied this theory to achieve synchronization between two identical unified chaotic systems. Passivity-based nonlinear controllers were obtained in [18, 19] to synchronize between two identical chaotic systems and between two different chaotic systems, respectively.

In [20], Huang et al. applied the passivity theory to investigate the hybrid synchronization of a hyperchaotic Lü system, but their method was based on exactly knowing the systems structure and parameters. In practical situations, some or all of the systems parameters cannot be exactly known in priori. Therefore, it is necessary to consider hybrid synchronization of hyperchaotic systems in the presence of uncertain parameters. In this paper, we apply the passivity theory to investigate the adaptive hybrid synchronization problem of a new hyperchaotic system with uncertain parameters.

## 2. Brief Introduction of the Passivity Theory

 Consider a nonlinear system modeled by the following ordinary differential equation:

(2) $$\begin{array}{c} \dot{x}=f\left(x\right)+g\left(x\right)u,\\ y=h\left(x\right), \end{array}$$

where *x* ∈ *R* ^*n*^ is the state variable; *u* ∈ *R* ^*m*^ and *y* ∈ *R* ^*m*^ are input and output values, respectively. *f*(*x*) and *g*(*x*) are smooth vector fields and *h*(*x*) is a smooth mapping. Suppose that the vector field *f* has at least one equilibrium point. Without loss of generality, one can assume that the equilibrium point is *x* = 0. If the equilibrium point is not at *x* = 0, the equilibrium point can be shifted to *x* = 0 by coordinate transform.

**Definition 1 (see [21])**

System (2) is a minimum phase system if *L*_*g*_ *h*(0) is nonsingular and *x* = 0 is one of the asymptotically stabilized equilibrium points of *f*(*x*).

**Definition 2 (see [13])**

System (2) is passive if there exists a real constant *β* such that for for all *t* ≥ 0, the following inequality holds:

(3) $$\begin{array}{c} \int_{0}^{t}u^{T}\left(\tau \right)y\left(\tau \right)d\tau \geq \beta , \end{array}$$

or there exists a *ρ* ≥ 0 and a real constant *β* such that

(4) $$\begin{array}{c} \int_{0}^{t}u^{T}\left(\tau \right)y\left(\tau \right)d\tau +\beta \geq \int_{0}^{t}\rho y^{T}\left(\tau \right)y\left(\tau \right)d\tau . \end{array}$$

If system (2) has relative degree [1,…, 1] at *x* = 0 (i.e., *L*_*g*_ *h*(0) is nonsingular) and the distribution spanned by the vector field *g*_1_(*x*),…, *g*_*m*_(*x*) is innovative, then it can be represented as the following normal form:

(5) $$\begin{array}{c} \dot{z}=f_{0}\left(z\right)+p\left(z,y\right)y,\\ \dot{y}=b\left(z,y\right)+a\left(z,y\right)u, \end{array}$$

where *a*(*z*, *y*) is nonsingular for any (*z*, *y*).

**Theorem 3 (see [13])**

If system (2) is a minimum phase system and has relative degree [1,1,…] at *x* = 0, then system (5) will be equivalent to a passive system and will be asymptotically stable at any equilibrium points through the following local feedback control:

(6) $$\begin{array}{c} u=a{\left(z,y\right)}^{-1}\left[-b^{T}\left(z,y\right)-\frac{\partial W\left(z\right)}{\partial z}p\left(z,y\right)-\alpha y+v\right]. \end{array}$$

## 3. Hybrid Synchronization of the New Hyperchaotic System

 Let system (1) be the drive system, and the response system is given by the following form:

(7) w˙1=a(w2−w1)+w4,w˙2=cw2−w1w3+u1,w˙3=−bw3+w1w2,w˙4=dw1+kw2w3+u2,

where *a*, *b*, *c*, *d*, and *k* are unknown parameters; *u*_1_ and *u*_2_ are controllers to be determined.

To investigate the hybrid synchronization, we define the state errors between the drive system (1) and the response system (7) as

(8) $$\begin{array}{c} e_{1}=w_{1}+x_{1},\\ e_{2}=w_{2}+x_{2},\\ e_{3}=w_{3}-x_{3},\\ e_{4}=w_{4}+x_{4}. \end{array}$$

Then the following error dynamical system can be obtained

(9) e˙1=a(e2−e1)+e4,e˙2=ce2−e1e3+x1e3−x3e1+u1,e˙3=−be3+e1e2−x1e2−x2e1,e˙4=de1+k(e2e3−x2e3+x3e2)+u2.

Let *z*_1_ = *e*_1_, *z*_2_ = *e*_3_, *y*_1_ = *e*_2_, and *y*_2_ = *e*_4_; the error dynamical system (9) can be rewritten as

(10) z˙1=a(y1−z1)+y2,z˙2=−bz2+z1y1−x1y1−x2z1,y˙1=cy1−z1z2+x1z2−x3z1+u1,y˙2=dz1+k(y1z2−x2z2+x3y1)+u2,

which is a normal formal

(11) $$\begin{array}{c} \dot{z}=f_{0}\left(z\right)+p\left(z,y\right)y,\\ \dot{y}=b\left(z,y\right)+a\left(z,y\right)u, \end{array}$$

where *z* = [*z*_1_, *z*_2_]^*T*^,  *y* = [*y*_1_, *y*_2_]^*T*^ and

(12) $$\begin{array}{c} f_{0}\left(z\right)=\left[\begin{array}{c} -az_{1}\\ -x_{2}z_{1}-bz_{2} \end{array}\right],\\ p\left(z,y\right)=\left[\begin{array}{cc} a & 1\\ z_{1}-x_{1} & 0 \end{array}\right],\\ b=\left[\begin{array}{c} cy_{1}-z_{1}z_{2}+x_{1}z_{2}-x_{3}z_{1}\\ dz_{1}+k\left(y_{1}z_{2}-x_{2}z_{2}+x_{3}y_{1}\right) \end{array}\right]. \end{array}$$

**Theorem 4**

The error dynamical system (9) is a minimum phase system.

**Proof**

Choose the following storage function:

(13) V(z,y)=W(z)+12yTy+12(a1−a)2+12(b1−b)2 +12(c1−c)2+12(d1−d)2+12(k1−k)2,

where *W*(*z*) = (*N* ^2^/4*ab*)*z*_1_^2^ + (1/2)*z*_2_^2^ is a Lyapunov function of *f*_0_(0), *N* is a bound of *x*_2_, namely, |*x*_2_ | ≤*N*, and *a*_1_, *b*_1_, *c*_1_, *d*_1_, and *k*_1_ are estimated values of the uncertain parameters *a*, *b*, *c*, *d*, and *k*, respectively.

The zero dynamics of system (11) describes the internal dynamics, which is consistent with the external constraint *y* = 0, that is, $\dot{z}=f_{0}(z)$, then we have

(14) ddtW(z)=∂W(z)∂zf0(z)=−N22bz12−bz22−x2z1z2=−b(z2+x22bz1)2+x224bz12−N22bz12≤−b(z2+x22bz1)2−N24bz12≤0.

Then, *f*_0_(*z*) is globally asymptotically stable. Meanwhile, $L_{g}h(0)=\left[\begin{array}{cc} 1 & 0\\ 0 & 1 \end{array}\right]$ is nonsingular. In the light of Definition 1, system (9) is a minimum phase system.

**Theorem 5**

If we choose the controllers as

(15) u1=−(c1+α)y1+(x3−N22b)z1+v1,u2=−(d1+N22ab)z1−k1(y1z2+x3y1−x2z2)−αy2+v2,

and the parameter estimation update laws as

(16) $$\begin{array}{c} {\dot{a}}_{1}=0,\\ {\dot{b}}_{1}=0,\\ c_{1}=y_{1}^{2},\\ d_{1}=z_{1}y_{2},\\ k_{1}=\left(w_{2}w_{3}+x_{2}x_{3}\right)y_{2}, \end{array}$$

where *v* = [*v*_1_, *v*_2_]^*T*^ is an external signal vector which is connected with the reference input, the error dynamical system (9) will be asymptotically stable at any desired equilibrium points with different values of *v*, and the hybrid synchronization between the two hyperchaotic systems (1) and (7) with different initial values will be achieved.

**Proof**

Taking the time derivative of *V*(*z*, *y*) along the trajectory of the error dynamical system (9) yields

(17) ddtV(z,y)=∂W(z)∂zz˙+yTy˙+(a1−a)a˙1+(b1−b)b˙1 +(c1−c)c˙1+(d1−d)d˙1+(k1−k)k˙1=∂W(z)∂zf0(z)+∂W(z)∂zp(z,y)y+yTb(z,y) +yTa(z,y)u+(a1−a)a˙1+(b1−b)b˙1 +(c1−c)c˙1+(d1−d)d˙1+(k1−k)k˙1.

According to Theorem 4, the error dynamical system (9) is a minimum phase system, that is, (∂*W*(*z*)/∂*z*)*f*_0_(*z*) ≤ 0, then (17) becomes

(18) ddtV(z,y)≤∂W(z)∂zp(z,y)y+yTb(z,y)+yTa(z,y)u +(a1−a)a˙1+(b1−b)b˙1+(c1−c)c˙1 +(d1−d)d˙1+(k1−k)k˙1.

Substituting (15) and (16) into (18) yields

(19) $$\begin{array}{c} \frac{d}{dt}V\left(z,y\right)\leq -\alpha y^{T}y+v^{T}y. \end{array}$$

Then, taking integration on both sides of (19), we get

(20) V(z,y)−V(z0,y0)≤−∫0tαyT(τ)y(τ)dτ +∫0tvT(τ)y(τ)dτ.

For *V*(*z*, *y*) ≥ 0, let *V*(*z*_0_, *y*_0_) = *μ*; the above inequality can be rewritten as

(21) ∫0tvT(τ)y(τ)dτ+μ≥∫0tαyT(τ)y(τ)dτ+V(z,y)≥∫0tαyT(τ)y(τ)dτ.

According to Definition 2, system (9) is a passive system. Because *W*(*z*) is radially unbounded, it follows from (13) that *V*(*z*, *y*) is also radially unbounded, so that the closed-loop system is bounded state stable for [*z* ^*T*^, *y* ^*T*^]^*T*^. This means that we can use the controllers (15) and parameter estimation update laws (16) to regulate the error dynamical system (9) to the equilibrium points, and the two hyperchaotic systems (1) and (7) with different initial values will be synchronized.

## 4. A Numerical Simulation

 In this section, a numerical simulations is carried out to verify the theoretical results obtained in Section 3. In the following numerical simulation, the fourth order Runge-Kutta method is applied to solve the equations with time step size 0.001. The system parameters are selected as *a* = 35, *b* = 3, *c* = 12, *d* = 1, and *k* = 0.5, so that system (1) can exhibit a hyperchaotic attractor.

**Figure 2:**
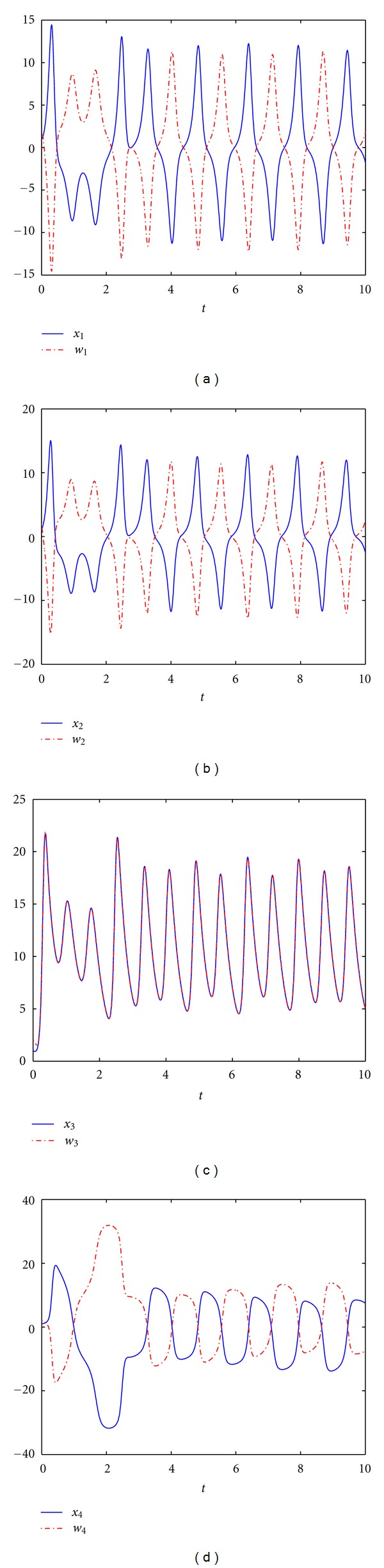
The time response of states for the drive system (1) and the response system (7).

**Figure 3:**
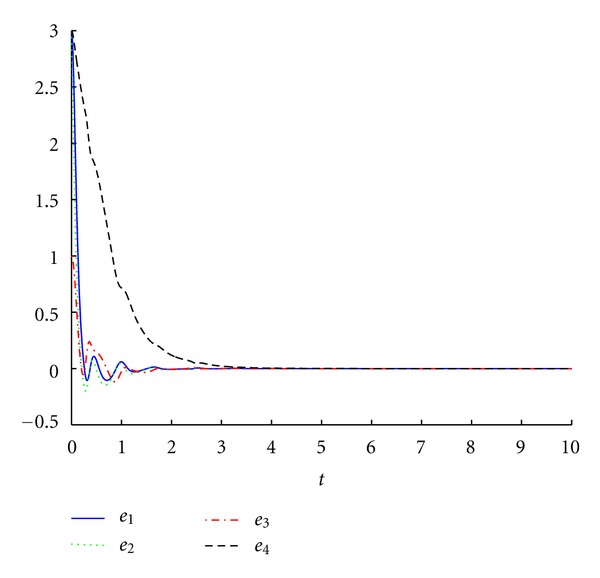
The time response of error states for the error dynamical system (9).

**Figure 4:**
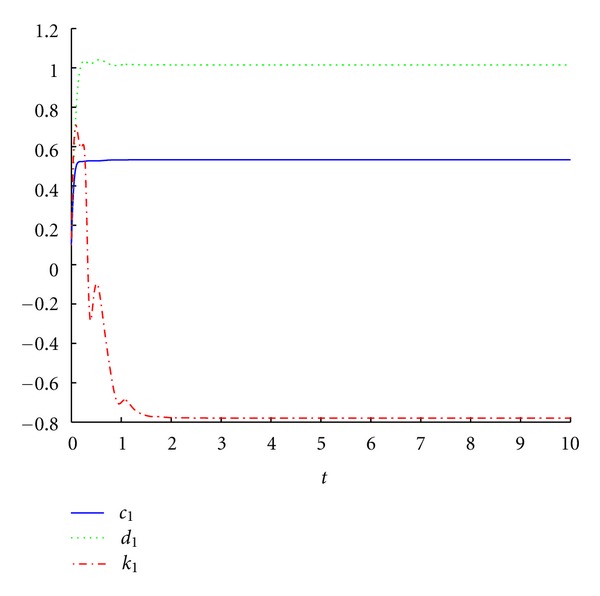
The estimations of the parameters *c*_1_, *d*_1_, *k*_1_.

 For the hybrid synchronization of the new hyperchaotic system, we consider the drive system (1) and the response system (7). The initial values for them are given as *x*_1_(0) = 1, *x*_2_(0) = 1, *x*_3_(0) = 1, *x*_4_(0) = 1, and *w*_1_(0) = 2, *w*_2_(0) = 2, *w*_3_(0) = 2, *w*_4_(0) = 2, respectively. Thus, the initial errors are *e*_1_(0) = 3, *e*_2_(0) = 3, *e*_3_(0) = 1, *e*_4_(0) = 3. And the initial values of the parameter estimation update laws are *a*_1_(0) = *b*_1_(0) = *c*_1_(0) = *d*_1_(0) = *k*_1_(0) = 0.1. We choose *α* = 1 and *v*_1_ = *v*_2_ = 0.  Figure 2 shows the time response of states determined by the drive system (1) and the response system (7) with the controllers (15) and the parameter estimation update laws (16). Figures 2(a), 2(b), and 2(d) illustrate antisynchronization of *x*_1_ versus *w*_1_, *x*_2_ versus *w*_2_, and *x*_4_ versus *w*_4_, and Figure 2(c) illustrates complete synchronization of *x*_3_ versus *w*_3_. As expected, one can observe that the trajectories of the error dynamical system (9) are asymptotically stabilized at the equilibrium point *O*(0,0, 0,0), as illustrated in Figure 3. From Figures 2 and 3, we can conclude that the hybrid synchronization between the drive system (1) and the response system (7) starting from different initial values is achieved. And the estimations of the parameters are shown in Figure 4, which converge to constants as time goes. 

## 5. Conclusions

 In this paper, we have investigated the adaptive hybrid synchronization of a new hyperchaotic system with unknown parameters, which includes complete synchronization and antisynchronization. Based on the passivity theory and the adaptive control theory, hybrid synchronization between two identical hyperchaotic systems with uncertain parameters starting from different initial values is achieved. A numerical simulation is presented to illustrate and verify the theoretical analysis. The simulation result and the theoretical analysis agree quite well.
